# Conveying Equipoise during Recruitment for Clinical Trials: Qualitative Synthesis of Clinicians’ Practices across Six Randomised Controlled Trials

**DOI:** 10.1371/journal.pmed.1002147

**Published:** 2016-10-18

**Authors:** Leila Rooshenas, Daisy Elliott, Julia Wade, Marcus Jepson, Sangeetha Paramasivan, Sean Strong, Caroline Wilson, David Beard, Jane M. Blazeby, Alison Birtle, Alison Halliday, Chris A. Rogers, Rob Stein, Jenny L. Donovan, ACST-2 study group, By-Band-Sleeve study group, Chemorad study group, CSAW study group, Optima prelim study group, POUT study group

**Affiliations:** 1 School of Social and Community Medicine, University of Bristol, Bristol, United Kingdom; 2 Nuffield Department of Orthopaedics, Rheumatology and Musculoskeletal Sciences, University of Oxford, Oxford, United Kingdom; 3 Rosemere Cancer Centre, Royal Preston Hospital, Preston, United Kingdom; 4 St George’s, University of London, London, United Kingdom; 5 Clinical Trials and Evaluation Unit, Bristol Royal Infirmary, School of Clinical Sciences, University of Bristol, Bristol, United Kingdom; 6 University College London Hospitals, London, United Kingdom; 7 National Institute for Health Research Collaboration for Leadership in Applied Health Research and Care West (NIHR CLAHRC West), University Hospitals Bristol NHS Trust, Bristol, United Kingdom; Stanford University, UNITED STATES

## Abstract

**Background:**

Randomised controlled trials (RCTs) are essential for evidence-based medicine and increasingly rely on front-line clinicians to recruit eligible patients. Clinicians’ difficulties with negotiating equipoise is assumed to undermine recruitment, although these issues have not yet been empirically investigated in the context of observable events. We aimed to investigate how clinicians conveyed equipoise during RCT recruitment appointments across six RCTs, with a view to (i) identifying practices that supported or hindered equipoise communication and (ii) exploring how clinicians’ reported intentions compared with their actual practices.

**Methods and Findings:**

Six pragmatic UK-based RCTs were purposefully selected to include several clinical specialties (e.g., oncology, surgery) and types of treatment comparison. The RCTs were all based in secondary-care hospitals (*n =* 16) around the UK. Clinicians recruiting to the RCTs were interviewed (*n =* 23) to understand their individual sense of equipoise about the RCT treatments and their intentions for communicating equipoise to patients. Appointments in which these clinicians presented the RCT to trial-eligible patients were audio-recorded (*n =* 105). The appointments were analysed using thematic and content analysis approaches to identify practices that supported or challenged equipoise communication. A sample of appointments was independently coded by three researchers to optimise reliability in reported findings. Clinicians and patients provided full written consent to be interviewed and have appointments audio-recorded.

Interviews revealed that clinicians’ sense of equipoise varied: although all were uncertain about which trial treatment was optimal, they expressed different levels of uncertainty, ranging from complete ambivalence to clear beliefs that one treatment was superior. Irrespective of their personal views, all clinicians intended to set their personal biases aside to convey trial treatments neutrally to patients (in accordance with existing evidence). However, equipoise was omitted or compromised in 48/105 (46%) of the recorded appointments. Three commonly recurring practices compromised equipoise communication across the RCTs, irrespective of clinical context. First, equipoise was overridden by clinicians offering treatment recommendations when patients appeared unsure how to proceed or when they asked for the clinician’s expert advice. Second, clinicians contradicted equipoise by presenting imbalanced descriptions of trial treatments that conflicted with scientific information stated in the RCT protocols. Third, equipoise was undermined by clinicians disclosing their personal opinions or predictions about trial outcomes, based on their intuition and experience. These broad practices were particularly demonstrated by clinicians who had indicated in interviews that they held less balanced views about trial treatments. A limitation of the study was that clinicians volunteering to take part in the research might have had a particular interest in improving their communication skills. However, the frequency of occurrence of equipoise issues across the RCTs suggests that the findings are likely to be reflective of clinical recruiters’ practices more widely.

**Conclusions:**

Communicating equipoise is a challenging process that is easily disrupted. Clinicians’ personal views about trial treatments encroached on their ability to convey equipoise to patients. Clinicians should be encouraged to reflect on personal biases and be mindful of the common ways in which these can arise in their discussions with patients. Common pitfalls that recurred irrespective of RCT context indicate opportunities for specific training in communication skills that would be broadly applicable to a wide clinical audience.

## Abstract 2

In a qualitative synthesis from six randomized controlled trials, Leila Rooshenas and colleagues examine how clinicians convey equipoise during recruitment for clinical trials.

## Author Summary

**Why Was This Study Done?:**

- Randomised controlled trials (RCTs) are a cornerstone of evidence-based medicine, but they need to recruit sufficient numbers of patients if they are to provide answers to the clinical questions they intend to address.
- Front-line clinicians are increasingly responsible for recruiting patients into RCTs, but there are questions around how they reconcile their own views about RCT treatments with the requirement to convey “equipoise” to patients (the principle that the patient would be neither advantaged nor disadvantaged if they were to receive any of the trial treatments under investigation).
- Clinicians’ attempts to communicate equipoise have not yet been investigated in the context of real doctor–patient interactions, and there are no evidence-based recommendations for how clinicians should convey equipoise to patients who are eligible for RCT participation.

**What Did the Researchers Do and Find?:**

- We aimed to investigate how clinicians attempted to communicate equipoise to patients across six RCTs, with the objectives of (i) identifying broad practices that support or hinder equipoise communication and (ii) examining how these practices compare with clinicians’ intentions.
- We interviewed clinicians (*n =* 23) to understand their intentions for communicating equipoise to patients, and audio-recorded their discussions (“appointments”) with RCT-eligible patients (*n =* 105) to investigate their actual practices.
- Despite clinicians’ best intentions, equipoise communication was omitted or undermined in almost half of the appointments (*n =* 48) through commonly recurring practices that were apparent across the RCTs, including (i) offering treatment recommendations, (ii) providing imbalanced descriptions of trial treatments, and (iii) disclosing personal predictions about RCT outcomes.

**What Do These Findings Mean?:**

- Communicating equipoise to patients is a delicate process that can be overridden or undermined through commonly recurring practices that emerge irrespective of clinical context.
- There are opportunities to create generic guidance for clinicians based on these common challenges, with a view to facilitating the communication of equipoise to RCT-eligible patients.
- Our findings were limited by our intentional focus on RCTs that were anticipated to experience recruitment difficulties, and thus this issue needs further consideration in RCTs that are deemed less challenging to recruit patients into.

## Introduction

Optimising recruitment to randomised controlled trials (RCTs) is a priority for clinicians, funding bodies, and healthcare decision-makers. Increasingly constrained resources reinforce the need for efficient generation of high-quality evidence to optimise patient care and inform service provision. Despite stringent review processes, at least half of funded RCTs fail to meet recruitment targets [1], and recruitment difficulties remain the most frequent reason for premature trial closure [2]. Not only are these difficulties associated with considerable financial waste [3], but poor recruitment can delay progress or leave unanswered clinical questions in underpowered or incomplete studies.

Attempts to unravel RCT recruitment difficulties have focused on the pivotal role of front-line clinicians as recruiters. Clinicians are on the interface of research and practice, and are often responsible for presenting the option of RCT participation to eligible patients. Clinicians’ perceptions of “equipoise” has repeatedly been cited as an assumed barrier to RCT recruitment [4,5]. Equipoise is broadly understood as a state of “substantial uncertainty” around treatment superiority [6], though there are discordant views around the degree and nature of uncertainty needed to justify randomisation [6]. Clinicians’ personal experiences and awareness of anecdotal or non-RCT evidence can disrupt their sense of equipoise. This has underpinned commentaries on why recruiting to RCTs can be so challenging—particularly in disciplines that have a propensity for swift and clear-cut decision-making (e.g., surgery) [7]. Equipoise at an individual clinician level (“individual equipoise”) can thus be hard to achieve—especially where ingrained clinical practices are under evaluation. The concept of “community equipoise” (or “clinical equipoise”) refers to the principle that there is no consensus in the *clinical community* about which treatment is best. In theory, this framing of equipoise enables clinicians to align their instinct to act in the individual patient’s interest with the utilitarian endeavour of benefiting future populations [8]. Although this viewpoint has been critiqued [9], clinical equipoise is widely accepted as the fundamental prerequisite that justifies an RCT. The pragmatic definition of equipoise adopted in this study framed the concept as a state where patients would be neither “advantaged nor disadvantaged” if they were to receive any of the trial treatments (as far as existing evidence would dictate) [10]. The recruiting clinician’s role was to convey this notion to eligible patients.

Previous research suggests that patients’ reasons for declining trial participation are often based on their interpretations of clinicians’ treatment preferences [11–14]; this raises questions about how successfully equipoise is conveyed to patients in practice. Interview studies have shown that clinical recruiters are not necessarily in individual equipoise [12,15–17] but assume that their personal views can be set aside during encounters with trial-eligible patients [4,18]. Although there has been extensive theoretical commentary on these issues [5,19,20], equipoise communication has not yet been investigated in practice. There is therefore little empirical evidence to inform practical guidance for clinicians, despite recognition that discussing equipoise is a core component of their roles. We set out to investigate how clinicians conveyed equipoise during recruitment appointments in ongoing RCTs, with a view to identifying practices that supported or hindered equipoise communication. A secondary objective of the study was to examine how clinicians’ practices compared with their reported intentions.

## Methods

Ethical approval for this study was granted by six UK research ethics committees, with full details (e.g., dates, reference numbers) presented in S1 Text.

This was a qualitative study that adopted ethnographic approaches. Data were available from three sources: (i) audio-recorded appointments in which clinicians presented RCTs to eligible patients (“recruitment appointments”); (ii) interviews with recruiting clinicians in which perceptions of equipoise about trial treatments were explored, to enable comparison of reported intentions and actual practices; and (iii) documentary analysis of trial protocols containing the scientific evidence underlying the RCT, to aid interpretation of observed practices (e.g., assessing accuracy of information provision).

### Context and Data Sources

Data from 16 publicly funded UK RCTs were available for inclusion, with an eventual sample of six selected for the study (see below). The sampling frame of 16 RCTs spanned a range of specialties (e.g., orthopaedics, vascular medicine, oncology). All were set in UK National Health Service–funded hospitals and had experienced (or were anticipated to experience) recruitment challenges. Data collection and analysis for the present study were conducted by the Qualitative Research Integrated within Trials (QuinteT) research team [21]. The lead of the QuinteT team (J. L. D.) was a collaborator or co-applicant on the RCTs, contributing an embedded methodological intervention that aimed to optimise recruitment: the QuinteT Recruitment Intervention (QRI) [22]. Members of the research team had no direct involvement in investigating the scientific questions the RCTs were addressing, but worked on the QRI. The QRI comprised an in-depth investigation of the RCT’s recruitment processes and sources of difficulty (Phase 1), followed by implementation of tailored strategies to address these challenges (Phase 2). Given this study’s focus on clinicians’ naturalistic practices, this paper draws exclusively on data collected prior to any intervention (i.e., Phase 1).

More than half of the RCTs in the sampling frame included surgical interventions (10/16). This reflected the team’s tendency to integrate the QRI into RCTs that were deemed “difficult” for recruitment by the RCTs’ chief investigators and funding bodies. RCTs with surgical interventions are often thought to pose recruitment challenges due to cultural issues and assumed difficulties in negotiating equipoise [7].

The QRI involved audio-recording recruitment appointments and interviewing recruiters to understand recruitment processes. The methods employed for audio-recording appointments and conducting interviews were the same for each RCT, as summarised below.

### Audio-Recording Appointments

Research nurses, local lead investigators, and central clinical trials units distributed information sheets about the recording process to recruiters via email/in person and obtained written consent for all of their recruitment appointments to be recorded (subject to patients’ permission). Research nurses and recruiters identified patients who were eligible to participate in each RCT and distributed information sheets about the audio-recording process in person/via post. Patients’ written consent was obtained in advance of the first appointment. Where prior contact was not possible, verbal consent was sought to record the first appointment; this recording was deleted if written consent was not subsequently provided. All appointments took place in one of 16 National Health Service secondary care hospitals around the UK. Audio-recordings were periodically transferred to the research team via encrypted data storage devices or secure file transfer.

### Interview Processes

Recruiters in each RCT were invited to take part in a semi-structured interview to establish their individual perceptions of equipoise around the trial treatments. All recruiters in the smaller-scale RCTs were invited to participate in this study. In larger multi-centre RCTs (e.g., with 20 centres or more), a purposeful mix of recruiters from centres that were recruiting well and centres that were struggling to recruit were invited for interview. Research nurses, local investigator leads, and central clinical trials units identified recruiting clinicians, distributed information sheets via email/in person, and passed on consenting individuals’ contact details to the interviewers (L. R., D. E., M. J., S. P., and C. W.). Although recruitment was also undertaken by research nurses in some centres, the present study focused exclusively on clinical recruiters in light of the study’s aims.

A standard topic guide was used to ensure key issues were consistently covered in interviews across the RCTs. This topic guide was modified as interviews progressed in each RCT to include emerging recruitment issues that were specific to that trial. The broad topics of equipoise and views about trial treatments were consistently covered across all interviews. Interviews took place over the telephone or on hospital premises, involved just the researcher and clinician participant, and were audio-recorded with permission. Most interviews were conducted prior to receipt of the recruiter’s audio-recorded appointments, though there were a few cases where recording of appointments had already begun at the time of interview. In these cases, the interviewers did not discuss any of their insights from listening to the appointments and recruiters had not received any feedback or training on their practices based on the recordings. All participants provided written informed consent. Field notes were used to record contextual details that might have influenced interview conduct and ideas for addition to topic guides.

### Sampling Criteria for Cross-RCT Investigation

Sampling for the present investigation involved (i) selecting the RCTs and (ii) selecting the audio-recorded appointments. A purposeful sample of four (of 16) RCTs was initially selected. Sampling was directed by the intention to include a range of clinical specialties and types of treatment comparison (e.g., trials comparing two or more existing treatments, trials comparing current practice with a new technology). Although the sampling frame included more RCTs with surgical interventions, we intentionally included two non-surgical RCTs to increase variation. Sampling was also partially informed by practical issues such as the number of audio-recorded appointments collected. Two further RCTs were later added to the sample to confirm/disconfirm emerging findings. Details of the RCTs are shown in Table 1. The balance of surgical to non-surgical RCTs in the final sample was reflective of the sampling frame.

**Table 1:** RCT details and associated number of appointments recorded (shown by participating clinician).

RCT Number Used in This Study (ISRCTN Reference)	Clinical Specialty	Description of RCT Comparison Groups	Clinician Identifier	Number of Recorded Appointments
RCT1 [23] (ISRCTN00786323)	General surgery	• Group 1: Surgical procedure 1 • Group 2: Surgical procedure 2	R1	19
			R2	10
			R3	16
			R4	8
RCT2 [24] (ISRCTN98387754)	Oncology	• Group 1: Adjuvant treatment • Group 2: Surveillance (with treatment offered on signs of cancer recurrence, if appropriate)	R5	6
			R6	2
			R7	2
RCT3 [25] (ISRCTN42400492)	Oncology	• Group 1: Adjuvant treatment • Group 2: Prognostic-test-directed adjuvant treatment	R8	4
			R9	7
			R10	2
			R11	1
RCT4 [26] (ISRCTN21144362)	Vascular medicine	• Group 1: Surgical treatment • Group 2: Stenting	R12	4
			R13	1
			R14	1
			R15	3
RCT5 [27] (ISRCTN33864128)	Orthopaedics	• Group 1: Arthroscopy with surgical manipulation • Group 2: Arthroscopy alone • Group 3: Active monitoring with specialist reassessment	R16	3
			R17	1
RCT6 [28] (ISRCTN89052791)	Oncology/surgery	• Group 1: Neoadjuvant treatment and surgery • Group 1: Definitive non-surgical treatment	R18	5
			R19	4
			R20	2
			R21	1
			R22	1
			R23	2

Audio-recorded appointments from the six RCTs were eligible for inclusion if they took place prior to any feedback to or training of the clinician (based on the recorded appointments), and if they were led by a clinician who had also been interviewed.

A total of 105 recruitment appointments met the study inclusion criteria and were analysed, coupled with 23 interviews with the clinicians who led these appointments. All clinicians were secondary care consultants (surgeons, oncologists, and neurologists). Interviews lasted between 21 min and 92 min. The selected data collated for this study were collected between October 2010 and December 2014. None of the 23 clinicians or 105 patients withdrew from the study (i.e., following provision of consent for the interviews or audio-recorded appointments).

The distribution of appointments per trial and clinician is shown in Table 1. In all, 83 of 105 appointments were with patients who ultimately declined trial participation (79%), 19 with patients who agreed to randomisation (18%), and three with patients where the outcome was not known (3%).

### Analysis

Analysis was conducted after all the data had been collected from the individual RCTs and was led by the corresponding author (L. R.), who had no involvement in the majority of the RCTs included in the sample (5/6). As such, analysis was primarily conducted by an “independent” researcher, who had no prior knowledge of links between the interviews with clinicians and their associated audio-recorded appointments. Analysis proceeded in three stages. First, analysis of audio-recorded recruitment appointments focused on how equipoise was conveyed in practice. This occurred in conjunction with reviewing the RCT protocols to understand the nature of equipoise underpinning each trial. Second, interview transcripts were analysed once initial analysis of appointments was complete. This allowed themes emerging from the audio-recorded appointments to be generated as inductively as possible, without being influenced by knowledge of clinicians’ views and perspectives. Third, findings from interviews and audio-recorded appointments were compared to examine how reported intentions corresponded with actual practice.

Recruitment appointments were listened to in full, and any discussion about the trial and/or trial treatments was transcribed verbatim if a full transcript of the appointment was not available. Full transcripts were available for four of the six RCTs, with two RCTs having “targeted transcription”. Material that was not transcribed verbatim included discussion related to “rapport building”, appointment scheduling, aspects of history taking not relevant to the diagnosis or trial, and technical descriptions of procedures that were often consistent across appointments for a particular recruiter. Transcripts were analysed using thematic techniques, guided by constant comparison methods derived from grounded theory methodology [29]. This involved line by line coding and organisation of codes into themes as analysis progressed. A coding frame was inductively developed, although there were a priori interests in examining how equipoise was communicated. The broad definition of equipoise provided by Djulbegovic et al. [10] informed coding. For example, any discussion/practices that supported the idea that the patient would be neither advantaged nor disadvantaged if they were to receive any/either of the trial treatments was coded as an example of conveying equipoise. Discussion/practices that did not support the above idea were coded as practices that challenged equipoise communication. Analysis was iterative in that previously coded transcripts were revisited in light of new codes/themes that emerged from later recordings. Analysis initially focused on RCT1, which had yielded the largest number of recorded appointments provided by the largest number of recruiters. Appointments in three other RCTs were subsequently analysed to confirm, challenge, and develop themes from RCT1. This was followed by analysis of two further RCTs to ensure saturation had been achieved. Saturation was defined as the point at which analysis of appointments from three consecutive RCTs resulted in no new themes or lines of enquiry. There was scope to continue sampling appointments from the 16 RCTs, but saturation was achieved once 105 appointments had been analysed from six RCTs.

Data from audio-recorded appointments were summarised in a matrix displaying each appointment (rows) against major themes from the final coding framework (columns). Rows were grouped according to clinician and RCT to facilitate comparison at three levels: (i) across a single clinician’s appointments to identify typical practices, (ii) between different clinicians of the same RCT to identify within-trial patterns and variations, and (iii) across trials to identify patterns and variations in practice across RCTs.

Interviews were transcribed in full and analysed using constant comparison methods, as described above. Data were organised and stored in NVivo (version 10, QSR International) to facilitate analysis. Sections of transcript that related to issues of uncertainty, equipoise, and clinicians’ views on the RCT treatments were coded in greater detail. Descriptive summaries of each clinician’s account from interview data were arranged alongside summaries of their observed practices to allow comparison.

A number of quality control processes were employed to ensure rigour in the reported findings. L. R. analysed all the data, with a subset of recordings from each trial double-coded by two independent members of the research team (D. E. and J. W.). Coding of individual transcripts was discussed in face-to-face meetings. Differences in interpretation were resolved through joint re-examination of original transcripts. Differences, where apparent, related to subtle misunderstanding of an RCT’s context (an isolated example) and researchers’ inclinations to occasionally code data in alignment with their particular research interests (which, on discussion, were generally deemed out of remit for this study). Any other differences related to individual researchers’ tendencies to code data at different levels (resulting in more or less depth) and differences in terminology. In the latter case, discussion in meetings reassured that this did not reflect different semantics. Findings were presented in team meetings to ensure these resonated with researchers who originally worked on the QRI integrated into each RCT. There was a conscious effort to search for negative cases (examples that contradicted the broad themes emerging from the data). These are reported in the findings, where relevant.

## Results

Given the complex and novel approach to data collection and analysis of this study, the findings have been presented in an order that most clearly conveys the key messages to emerge from the study. A summary of clinicians’ individual perspectives on equipoise is presented first, to provide a framework for drawing comparisons between reported intentions and actual practices. The findings from the audio-recorded appointments—the focus of this study—are presented next. References are made to clinicians’ interview accounts where relevant to demonstrate how reported intentions compared with actual practices.

### Interview Findings: Clinicians’ Individual Perceptions of Equipoise

**Fig 1:**
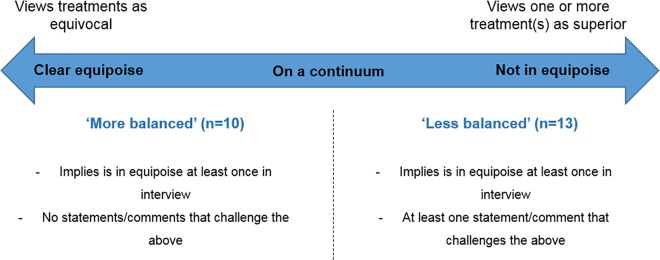
Clinicians’ individual perceptions of equipoise, based on interview accounts.

Most clinicians expressed commitment to the RCT, acknowledging uncertainty amongst the clinical community about how patients should be treated (community equipoise). All clinicians acknowledged the need for evidence and indicated that they were individually “uncertain” about optimal treatment. However, interview accounts revealed a spectrum of uncertainty, ranging from complete ambivalence to strong beliefs about the superiority of a particular treatment. On the basis of the congruence of these findings with previous research [4], clinicians could be considered to fall on a continuum ranging from “clear equipoise” (equivocal views about trial treatments) to “not in equipoise” (treatment preferences and/or unbalanced views about trial treatments). All clinicians reported being uncertain or “in equipoise” on at least one occasion during the interview, but approximately half presented treatment preferences or polarised views at other stages of the interview. Clinicians were therefore divided into two groups: “more balanced” and “less balanced” (Fig 1). Those categorised as more balanced (*n =* 10) consistently indicated that they viewed the trial treatments in equilibrium (Box 1). Less balanced clinicians (*n =* 13) expressed at least one clear preference or polarised opinion about treatment superiority during their interview—either in a general sense or in relation to particular sub-groups of eligible patients (Box 1). Evidence to support categorisation of each of the 13 less balanced clinicians as such is presented in S1 Data.

**Box 1. Interview Extracts Comparing More Balanced and Less Balanced Clinicians’ Views on RCT Treatments**

**More Balanced Group**

**R5**: Um, so in other words here equipoise, as I say I think I do have equipoise here because I really do not know which is the best treatment for a patient. (R5, RCT2—more balanced group)

**R1**: If I thought I knew what the answer was then I probably wouldn’t be interested in being in the trial. (R1, RCT1—more balanced group)

**R9**: I don’t know what results this is going to be, I think there remains a degree of uncertainty. It keeps me awake at night. (R9, RCT3—more balanced group)

**Less Balanced Group**

**R7**: [My] gut reaction is probably that it [treatment *x*] does something and something needs to improve the outcome of these patients really, but my normal practice is not to give [treatment *x*] because I have to do things that have got an evidence base. (R7, RCT2—less balanced group)

**R4**: I think we’ll see what other trials have demonstrated. I think we’ll see that [treatment *x*] is better initially. (R4, RCT1—less balanced group)

**R2**: I’m struggling personally with what we’re going to do in the trial with the patients who for whatever reason don’t get on with [treatment *y*]. (R2, RCT1—less balanced group)

Regardless of their individual perceptions of equipoise, all clinicians indicated that their aim was to inform patients about the RCT without betraying their personal views. This reflected these clinicians’ appreciation for community equipoise, which in turn reinforced prior work conducted in this field [4]. Although some anticipated that they might inadvertently influence patient choice, most believed they could achieve neutral information provision:

I can be unbiased during a presentation to them. [‖] If you want me to be part of the randomised trial then I can’t, I have to be completely non-biased. (R4, RCT1—less balanced group)

I’m probably biased towards a surgical treatment slightly, but I try not to express that. (R20, RCT6—less balanced group)

The less balanced versus more balanced categorisation provided a framework for comparing clinicians’ individual perceptions of equipoise with their practices during recruitment appointments.

### Audio-Recorded Appointment Findings: Articulating Equipoise

Equipoise issues were discussed in most of the observed appointments (83 of 105, 79%). Although the term “equipoise” was generally not used, the principle was described in a variety of ways. Equipoise was most often discussed in collective (“we”) terms (79 of 83 [95%] appointments), thus indicating a tendency for clinicians to convey community (rather than individual) equipoise during patient encounters. This included references to clinical communities not knowing which treatment is best and presentations of equipoise as a global phenomenon (“nobody knows which treatment is best”). Individual equipoise (“I don’t know which is best”) was expressed in only 27 of 83 appointments (33%), though this was always in conjunction with statements of collective equipoise.

Equipoise was most commonly portrayed through statements indicating an absence of knowledge about which treatment was best (e.g., “we don’t know what’s best”) or through explanations of what the RCT was seeking to achieve (e.g., “we’re trying to find out”). Statements specifying uncertainty were also used, albeit less frequently (e.g., “we are uncertain”). Equipoise was also occasionally communicated by emphasising treatment equivalence, most often through reassurance that either/any trial treatment was equally appropriate for the patient. These statements were not mutually exclusive, with most appointments incorporating one or more of these statements (Table 2).

**Table 2:** Clinicians’ descriptions of the concept of equipoise to patients during RCT recruitment appointments.

Type of Statement	Examples	Appointments That Included at Least One Example of the Statement Type, *n =* 83	
		Number	Percent
Intentions of RCT: specification of what the RCT is seeking to achieve	“We’re trying to find the answer”; “we have a study that’s looking at comparing [treatment *x*] and [treatment *y*] to see which is better”	64	77%
Absence of knowledge: specification of not knowing	“We don’t know what’s best”; “I would love to know which is better”	61	74%
Limitations or absence of evidence	“There is not enough evidence to say”	28	34%
Uncertainty: statements that allude to not being sure or certain	“We do these studies when there is uncertainty”; “I can’t say for sure which is best”	27	33%
Emphasising patient suitability for both/all treatments	“My assessment of you is that you would be suitable for either treatment”	22	27%

### Audio-Recorded Appointment Findings: Difficulties Conveying Equipoise

Although equipoise was discussed in most appointments, over half of the clinicians (*n =* 16) encountered difficulties in consistently conveying equipoise throughout their discussions with patients. Most of these clinicians belonged to the less balanced group (*n =* 13), although there were three more balanced clinicians who also encountered difficulties.

Three recurring practices obstructed or obfuscated communication of equipoise. These practices varied in subtlety and were identified across multiple RCTs, irrespective of context. The patterns were: (i) overriding equipoise (e.g., by recommending a treatment), (ii) undermining equipoise (e.g., by communicating a preference), and (iii) omitting equipoise (e.g., avoiding presenting or discussing equipoise). Each of these is presented in more detail below. Overall, 48 of the 105 appointments (46%) demonstrated one or more of these difficulties.

#### Overriding equipoise

Equipoise was overridden in nine appointments by clinicians clearly recommending a treatment option. Five clinicians from five RCTs recommended treatments in at least one of their appointments with trial-eligible patients. Most of these recruiters (*n =* 4) had revealed discomfort with one or more of the trial treatments in their interviews. This discomfort was apparent in appointments when patients appeared unsure or ambivalent towards treatment options, or had particular characteristics that challenged that particular clinician’s sense of equipoise. For example, R4 (RCT1; Box 2) had expressed in the interview a preference for patients to receive particular trial treatments on the basis of their age, but also expressed the intention to refrain from conveying these biases to patients. Despite this, the recruiter went on to provide treatment recommendations to a number of patients, justifying their advice on the basis of these characteristics. Similarly, R8 (RCT3; Box 2) had intended to convey equipoise to the full range of eligible patients, yet reverted to recommending a particular treatment when faced with patients whose clinical characteristics placed them near the limits of the trial’s eligibility criteria (for the patient in R8’s extract in Box 2, the estimated level of benefit of treatment *x* placed the patient within the trial’s eligibility criteria; this RCT’s justification was built on the premise that there is uncertainty around the eligible patient group’s potential to benefit from treatment *x*).

**Box 2. Extracts Comparing Clinicians’ Views/Intentions for Communicating Equipoise as Reported in Interviews with their Practices of Providing Treatment Recommendations in Audio-Recorded Recruitment Appointments**

**R4, RCT1—Less Balanced Group**

**Clinician’s views/intentions expressed in interview:**

**R4**: Hmm, I have to say, I mean being honest, with the more elderly patients with more co-morbidities, diabetes, I tend to go straight for [procedure *x*] on those patients (in routine practice). Hmm, just because they’ve got a limited life expectancy. In the younger patients I get with the less co-morbidities, I certainly have a preference for [procedure *y*].

**R4**: I think you present it fairly and then leave it up to the patient to make their own mind up. [‖] I can’t then guide them into which one I think they should have.

Communication in practice:

**R4 [at outset of appointment]**: Have you thought about what sort of [treatment] you want?

**Patient**: Yes. [Patient explains preference for treatment *x*]

**R4**: Ok. I’m going to talk to you about that, because of your age. I’m going to have a chat with you‖ and the way you [explains characteristic]. I might try to steer you in a different direction I’m afraid. [‖] I’ll explain that a little bit more. Let me go through the options‖. I mean, once you’ve heard all the options, I’m going to ask you the same question and we’ll have a chat about it. [Recruiter explains treatment options]

**R4**: And that’s why I was going to ask you what do you think you should have done. Because with your age and [characteristic], you would do well with [treatment *y*]. [‖] My feeling would be for [treatment *y*], um—because you’re otherwise relatively fit. The only real illness you have is [names illness]. I’m just wary of putting someone so young through [treatment *x*].

**R8, RCT3—Less Balanced Group**

**Clinician’s views/intentions expressed in interview:**

**R8**: Then you start talking about the numbers and then at the end of the consultation you say, “Actually you can see the benefit from [treatment *x*] isn’t great, so normally this is a decision which is made by the patient and the doctor together.”

Communication in practice:

**R8**: [So I think] I mean if you think about it in a stepwise way, leaving the trial to one side, do you think that level of benefit is enough to warrant [treatment *x*]?

**Patient**: Yes.

**R8**: Yes [overlaps]. And I would say‖ certainly your doctors would agree with you, and most patients would agree with you. So I think, the initial question is, should we proceed with [treatment *x*]‖ the answer is “yes”.

#### Undermining equipoise: imbalances in treatment presentation, and disclosure of opinion

Another pattern of communication that was problematic was the more subtle undermining of equipoise. This was apparent through imbalanced descriptions of trial treatments, and disclosure of clinicians’ opinions and predictions about RCT findings. Fourteen clinicians (from all six RCTs) demonstrated at least one of these patterns. All bar one of these clinicians belonged to the less balanced group, based on the views they had expressed in interviews.

Equipoise could be undermined by implying that one trial treatment was more established than another. This was particularly problematic in RCTs evaluating treatments that were already used in routine practice. The treatments in these RCTs were particularly prone to clinical practice variations based on clinician preference. Some recruiters used loaded descriptors such as “gold standard” or “global standard” in relation to one trial treatment, without further explanation that the comparator may be just as good or better. These practices contrasted with other recruiters (from the same RCTs) who introduced the treatments in more neutral terms (e.g., by discussing practice variations or emphasising current assumptions that both/all trial treatments were equivalent). Within-trial variations in different recruiters’ descriptions of trial treatments are illustrated in Box 3. One of these quotes is from a more balanced clinician considered to be an outlier (R5), in that he/she did not follow the same pattern observed throughout this study. Whilst most of the more balanced clinicians did not demonstrate any of the difficulties in equipoise communication encountered by the less balanced clinicians, R5 was an exception. Despite being categorised as more balanced on the basis of the interview, this clinician went on to undermine equipoise in practice.

**Box 3. Communication in Practice: Extracts from Audio-Recorded Appointments Illustrating Clinicians’ Variable Descriptions of Treatments Available in Routine Practice**

**R7**: People historically have either done very close follow-up, because they’ve said, well look there’s no evidence to give [treatment *x*]. Or they’ve done [treatment *x*] because they said there’s no evidence not to give [treatment *x*]. (R7, RCT2—more balanced group)

**R5**: Well [‖] let me tell you what [‖] I think the global standard of care is. I think that, um‖ so nobody’s saying we would never give you [treatment *x*]. But we would not normally give [treatment *x*] at this point. (R5, RCT2—more balanced group; outlier)

**R20**: We’ve been very keen to try and compare the two treatments—certainly [treatment *x*] has always been held up as the gold standard, which is fair enough. (R20, RCT6—less balanced group)

**R23**: The difficulty is that there [‖] isn’t a right and a wrong way of treating it. There are two very different treatments available for it and at the moment we don’t really know whether one is better than another. (R23, RCT6—more balanced group)

Treatment superiority was also implicitly communicated in some clinicians’ descriptions of the research question. For example, some clinicians from RCT1 and RCT6 presented the trial as an investigation of whether treatment *x* was “as good as” treatment *y* (R4 and R20; Box 4). Both treatments from these RCTs were widely used (albeit variably) in routine practice. These statements and hypotheses did not reflect the information and evidence presented in the RCT protocols. Furthermore, these loaded descriptions were in stark contrast to more neutral descriptions of the treatments/RCT rationale provided by other clinicians recruiting to the same RCTs (e.g., R1 and R21; Box 4).

**Box 4: Communication in Practice: Extracts from Audio-Recorded Appointments Demonstrating Within-RCT Variation in Recruiters’ Descriptions of Trial Treatments**

**R4**: Part of this trial is to establish whether if the follow up for [treatment *y*] is good, is it as good as [treatment *x*]. (R4, RCT1—less balanced group)

**R1**: We don’t know if one [treatment] is better than the other or if they are just alternatives. (R1, RCT1—more balanced group)

**R20**: Whether [treatment *x*] has got a tiny benefit over the [treatment *y*] we don’t know, that’s what we are trying to find out. (R20, RCT6—less balanced group)

**R21**: Now, there is evidence—or the—people think there is reasonable evidence that both treatments have similar outcomes but we don’t know that for sure. There’s not been a trial to show if one treatment is better at curing you than another treatment. (R21, RCT6—more balanced group)

Patients’ responses provided insight into how recruiters’ descriptions of trial treatments could be interpreted (Box 5). Use of terms such as “gold-standard” and statements about one treatment being potentially “as good as” another were not supported by information stated in the RCT protocols. These descriptors had a clear influence on patients’ interpretations of treatment safety and likelihood of effectiveness (Box 5, extracts 1–3). Some terms could be considered “loaded” based on the clinical context in which they were mentioned. For example, some descriptions of tumour removal in RCT6 implied greater assurance of therapeutic benefit with one procedure over the other (Box 5, extract 4).

**Box 5. Communication in Practice: Extracts from Audio-Recorded Appointments Illustrating Patients’ Interpretations of Imbalanced Treatment Presentations**

**Extract 1: R18, RCT6—Less Balanced Group**

**R18**: And there have been some very good results when we do [treatment *x*], but they’ve never ever been compared with what has been, over many years, the gold standard treatment [which is treatment *y*].

**Patient [later]**: Right. Well, if I went into the study and obviously was offered [treatment *y*], and it didn’t work?

**Extract 2: R22, RCT6—Less Balanced Group**

**R22**: One is—it’s a traditional treatment which is practised mostly around the world—or the world most commonly—and also more traditionally is (treatment *x*).

**Patient partner [later]**: This has all coming about because of the new technology [treatment *y*] or whatever. I mean, if you’re talking thirty, forty years ago, automatically you’d have had [treatment *x*].

**Patient partner [later]**: But I’d just say I‖ do we want to try the new technology, or stick with the old technology?

**Extract 3: R4, RCT1—Less Balanced Group**

**R4 [after describing definite beneficial effects of treatment *x*]**: However, if you use [treatment *y*], well, potentially you can get the health benefits that you want, but it does require a lot of input from you.

**Patient**: Looking at what you told us, [treatment *x*] would be the best option for me, because it’s a quick answer to my health problems.

**Extract 4: R18, RCT6—Less Balanced Group**

**R18**: The first is [treatment *x*] to remove the cancer to give us the chance of cure.

**R18 [later]**: [Treatment *y*] is designed to shrink down the tumour and try and kill it off.

**Patient relative**: The thing that’s worrying me if she has [treatment *y*]‖ if that doesn’t take it away‖ but if she has [treatment *x*] then it’s gone.

Equipoise was also undermined by clinicians predicting RCT findings. This was demonstrated by half of the clinicians observed, mostly in the less balanced group. Some clinicians tempered their predictions about trial outcomes with uncertainty and reiteration of the trial aims:

**R7**: That’s because I don’t know whether giving [treatment *x*] now is any better than just closely following you up. My gut reaction is that it may be, but I can’t prove, until we finish off this study. (R7, RCT2—less balanced group)

There were, however, also examples of clinicians expressing their predictions and opinions without reiterating uncertainty:

**R5**: We would be reluctant to recommend [treatment *x*] in the absence of evidence because I think if there is a benefit from [treatment *x*] it will be small. (R5, RCT2—more balanced group; outlier)

Four of the RCTs included examples of clinicians not fully and accurately communicating equipoise as it was conveyed in the RCT protocols. Examples of practices that jeopardised equipoise communication included presenting non-evidence-based information with certainty and showing confidence in the likely outcome of the RCT. The extracts below compare two clinicians’ approaches to introducing equipoise in an RCT that compared two surgical interventions with conservative therapy (RCT5). The first clinician (R16) articulates the three-way uncertainty as per the trial protocol, whereas the second (R17) implies a degree of certainty that one treatment (surgery) works. Equipoise as communicated by R17 thus appeared to be about “how”, rather than “whether”, surgery was effective:

**R16**: The trouble for us at the moment is we don’t know whether to be offering people this operation. [‖] And, if it is to work, we don’t know quite how it works. (R16, RCT5—less balanced group)

**R17**: How that surgery works is not at all clear. What we’re doing at the moment is participating in a trial. The idea is that that should hopefully give us some information as to which of those forms of treatment is producing the improvement in the people who do get better. (R17, RCT5—less balanced group)

Over-expressing confidence in a particular trial arm without reiterating uncertainty was particularly evident in RCTs that compared current practice with novel approaches. Comparison of clinicians’ communication in practice (in appointments) with their expressed views/intentions (in interviews) revealed that tendencies to present opinions as facts were likely to be unwitting:

**R10 [in interview]**: So I say [to patients] because we don’t know that [novel technology] is useful, the way the trial works is that half the people will just get‖ [goes on to explain randomisation]. (R10, RCT3—less balanced group)

**R10 [in appointment]**: I have no concerns that it’s a dodgy [technology] or anything. I think it’ll give us a good result. (R10, RCT3—less balanced group)

#### Omitting equipoise

Discussion of equipoise was omitted in 22 appointments, from five of the six RCTs. Some of these omissions were associated with the issues discussed above (e.g., clinician-initiated treatment recommendations). The remainder were characterised by particular patterns in the ways clinicians structured or led the discussion: clinicians leading these appointments directly elicited patients’ preferences and accepted these without exploration, or geared the appointments towards patients selecting a treatment. These patterns were demonstrated by clinicians from both the less balanced (*n =* 3) and more balanced (*n =* 2) groups, and thus occurred irrespective of clinicians’ individual perceptions of equipoise. Some of these clinicians had, however, expressed particular areas of discomfort in their interviews: namely, difficulties around transitioning from routine practice to recruitment appointments (Box 6, R18 and R2) and concerns about how to deal with patient preferences without appearing coercive (Box 6, R22).

**Box 6. Interview Extracts Providing Insights into Why Clinicians Omit Equipoise**

**R18**: If you think about it we’re often expecting patients to get involved in decision-making, and in other situations outside of randomised studies we might say to the patient, “Look, there’s treatment A or there’s treatment B—which would you prefer?”, and then we’re expecting patients to decide. (R18, RCT6—less balanced group)

**R2 [in reference to routine practice]**: [When patients are] very clear about why they want to have an operation, or which operation they want to have, then I used to heave a sigh of relief because I thought, “Great, this patient is on board, they are clearly engaged.” [‖] So the problem of course now is that we have a situation whereby people are coming to us and we want them to say, “I was rather hoping you’d tell me the difference between the operations so that you can help me make my mind up,” because then you can say exactly “Actually wonderful, [‖] we don’t know which is the best operation.” [‖] So of course we actually have to, in a sense, undo what we’ve done. (R2, RCT1—less balanced group)

**R22**: If you won’t accept a patient’s choice then you’ve probably‖ you’re in the grounds of coercion. (R22, RCT6—less balanced group)

## Discussion

This study investigated how clinicians communicated equipoise in RCT recruitment appointments. Presenting and sustaining equipoise was found to be a challenging process that was highly entwined with clinicians’ own perceptions of equipoise. Clinicians varied in terms of their individual sense of equipoise, but were committed to the RCTs and reported that they intended to present treatments to patients in a neutral and balanced way. These expressed intentions did not always translate into practice—especially when clinicians harboured individual preferences or polarised views about treatments. Although equipoise was articulated in most appointments, observations across the RCTs revealed common practices that compromised equipoise communication. Clinicians with treatment preferences or less balanced views overrode or undermined equipoise through offering treatment recommendations, presenting imbalanced descriptions of trial treatments, or making clear their predictions about trial outcomes. These difficulties arose despite clinicians’ intentions to conceal or suspend their personal inclinations or beliefs.

A key strength of this study was its analysis of real (rather than reported) practices and events. Issues of recollection bias and selective reporting were thus minimised. Paired interview and observational data allowed novel comparison of intended and actual practices, providing potential explanations for some of the observed practices. The study drew on a large dataset derived from a range of RCTs and clinical contexts, and focused on common practices that were identified across these settings. As such, findings were not limited to a particular RCT context and are likely to be applicable more widely. A qualitative approach was crucial for this particular research question given the absence of prior empirical research and the need for an inductive approach. We made a conscious effort to look across a broad range of RCTs and focus on the practices that were common across these contexts. The reported findings are thus likely to be salient to clinicians across specialties.

The study had a number of limitations. Data were derived from RCTs that were experiencing (or were anticipated to encounter) recruitment difficulties. There was a prior intention to focus on challenging RCTs with a view to informing strategies to overcome recruitment difficulties, but our findings need to be considered further in RCTs that recruit well. Another limitation was the reliance on RCTs involving surgical interventions, which could have raised particular issues. Nonetheless, our sample also included RCTs that did not include surgical interventions, and although these were fewer in number, we did not identify any notable differences between RCT types in relation to the key findings. Integration of the recruitment sub-study (QRI) across multiple RCTs meant that some processes (e.g., study invite distribution) were organised centrally (e.g., by clinical trials units) or influenced by local issues (e.g., research nurses’ capacity and work patterns in RCT centres). We did not have a sense of how many patients and clinicians declined participation in our study (or were not approached), and as a consequence it is possible that the data captured provide insights from particular groups of patients/clinicians. We believe this is unlikely in the case of patients, as informal discussion with research nurses revealed that it was rare for patients to decline consent for audio-recording their appointments. If recordings were not captured, this tended to be due to logistical or organisation issues. However, findings may be limited by the self-selected clinicians who agreed to be interviewed and to have their appointments audio-recorded. As these individuals may have been particularly interested in improving communication or supportive of the RCT, it might be that problematic presentations of equipoise are more pronounced in appointments led by clinicians who might not have been as engaged with the research. The audio-recording process itself may have altered clinicians’ practices, though, again, any Hawthorne effect is likely to have manifested as recruiters being more mindful of concealing their views. Some of the clinicians participating in the study contributed only one appointment for observation, which limited the scope to compare clinicians’ reported and actual practices. The limitations relating to the lack of information about clinicians/patients who did not volunteer/agree to participate, and the potential influence of the Hawthorne effect, are partly countered by the fact that we identified similar challenges across clinicians/RCTs. This lends some assurance to the integrity of the key findings presented. Finally, like any interview-based study, it is possible that participants’ accounts were influenced by their knowledge of the researchers’ interests (in this case, recruitment). Clinicians were nonetheless not aware of our specific interest in investigating equipoise.

Much of the literature around clinical equipoise has been theoretical, although there have been some empirical studies that have focused on clinicians’ reported difficulties with negotiating equipoise. These studies indicate that clinicians often assume that personal struggles with equipoise can be set aside during patient encounters and have no bearing on recruitment [4,18]. However, studies reporting patient perspectives indicate that their decisions around trial participation can be guided by their interpretations of what their clinician prefers or recommends [11–14]. There is also evidence to suggest that patients construct these interpretations based on perceived cues, rather than direct statements [14]. The present study bridges these bodies of evidence by providing insight into what actually happened during particular RCT recruitment appointments. This has enabled a detailed examination of how equipoise is put into practice, and identification of commonly recurring ways in which equipoise communication can be compromised.

Some of our findings are supported by previous research involving observations of recruitment appointments. These studies were largely nested in single RCTs and reported on trial-specific challenges clinicians encountered. For example, the potential for loaded terminology to disrupt equipoise has been reported in RCTs of laryngeal and prostate cancer [30,31]. The scope to look across RCTs helped us to distinguish particular aspects of discussions that are prone to loaded terminology or misrepresentation, such as portrayal of current practice and descriptions of the RCT question [30]. These areas could be amenable to generic training or guidance that encourages clinicians to carefully articulate the information specified in the trial protocol.

Though there are few multi-RCT studies of communication practices, one study by Jenkins and colleagues included a content analysis of how frequently particular trial concepts were mentioned in recruitment appointments [32]. Although “equipoise” was not specifically considered, the authors reported that “uncertainty” was mentioned in almost all of the 82 appointments observed. By contrast, we found equipoise discussion was omitted in 20% of the observed appointments. This discrepancy may be explained by differences in sample sizes and clinical contexts. Jenkins and colleagues observed fewer clinicians’ practices (*n =* 5) and focused solely on oncology trials. Oncologists are more accustomed to discussing issues of equipoise and uncertainty, compared to clinicians working in other specialties, such as surgery [7]. The greater diversity of clinical specialties and greater number of clinicians observed (*n =* 23) in our study may explain the comparatively higher proportion of recruitment appointments that omitted discussion of equipoise/uncertainty. Other studies have also shown that equipoise is often omitted from RCT recruitment discussions, though these studies also specifically measured the frequency of statements of “uncertainty” [33,34]. Reported presentations of equipoise in this study were based on clinicians’ own terms, rather than preconceived assumptions. This confirmed that statements of equipoise often extend beyond mere mention of uncertainty, as suggested in prior descriptions of how clinicians discuss equipoise in interviews [35].

Introducing the concept of equipoise to patients can be a challenge in its own right, as indicated by our findings that some clinicians were inclined to omit the concept from recruitment appointments. The substantial literature on clinicians’ management of medical uncertainty may be particularly helpful for illuminating explanations for the omission of equipoise. Uncertainty is a complex, multifaceted phenomenon that is pervasive throughout the medical literature. The definition of equipoise used in this study most closely aligns with the notion of scientific ambiguity—uncertainty in clinical recommendations on the basis of insufficient scientific evidence [36]. There has been remarkably little research that investigates how scientific ambiguity is operationalised in routine practice, although the small body of evidence that does exist has highlighted two broad strategies adopted by clinicians: creating an illusion of certainty [37–39] and transferring uncertainty to others (such as the patient) [37,38,40,41]. Our observations of clinician-initiated recommendations and tendencies to gear appointments towards patients choosing a treatment may therefore reflect strategies used to address uncertainty in routine practice, and underlie why recruitment needs to be recognised as a different activity [42]. There are opportunities for future research to compare recruitment appointments with routine consultations to explore issues arising from these different contexts.

The concept of equipoise is poorly demarcated in the literature, and, as such, the findings of this study need to be interpreted in light of the assumptions we have made and the stances we have taken. Equipoise can be considered in two contexts: first, as a phenomenon that justifies an RCT proposal and, second, as a rationale for randomisation presented during an RCT recruitment appointment. This study assumed that public funding of the RCTs indicated there was sufficient justification for the RCTs, although questions have also been raised around whether equipoise should take account of considerations such as social values and cost of treatment [43]. Definitions of equipoise in the context of the RCT recruitment appointment can also vary. Rather than an inductive grounded theory investigation [29], we specified a priori a broad and pragmatic definition of equipoise as a state where a patient would be neither advantaged nor disadvantaged if they were to receive any of the RCT treatments under investigation, based on existing evidence. We assumed that a patient who is clinically eligible for an approved RCT should be equipped with evidence-based information that ensures they are aware that the interventions under consideration would be safe, equivalent, and appropriate for them. This does not, however, account for external or patient-related non-clinical factors that can alter this balance. Future research needs to examine how patients’ values, beliefs, and preferences are managed in the RCT recruitment appointment, and the implications this has for recruitment outcomes. Focusing on recruiters’ explanations and information provision in isolation risks creating an oversimplified picture of what is likely to be a complex set of interactions. Our findings may also have oversimplified some issues through our pragmatic labelling of clinicians as more or less balanced. This labelling acted as a proxy for individual equipoise but does not take account of the continuum of views we identified. Our findings should thus be viewed as an initial step, in need of future refinement.

The findings of this study have practical implications. Identification of commonly recurring communication challenges provides opportunities for targeted strategies to improve practice. The practices that obfuscated equipoise communication varied in nature and would require different solutions. Treatment recommendations were the most extreme practices that contradicted equipoise. Recommendations were often rationalised in terms of patients’ characteristics, presented by clinicians who had alluded to being uncomfortable with particular aspects of trial eligibility criteria. Previous research has also shown that clinicians’ perceptions of equipoise can waver at the limits of eligibility criteria [4]. Like any aspect of clinical practice, patients’ suitability for trial interventions needs to take into account individual patient circumstances. However, difficulties arise when patients are steered against RCT participation on the basis of clinical or biological characteristics that are not stated as formal exclusion criteria. Filtering patients on these bases can have implications for the generalisability of RCT findings and interfere with eligible patients’ autonomy to make decisions about trial participation. One solution to untangling these difficulties might be to promote open discussion about discomfort or concerns about trial eligibility criteria during trial initiation meetings and site visits [4,42]. The QRI uses evidence from audio-recorded appointments and interviews to air these issues during investigator feedback and training sessions [22]. Recording detailed reasons for patients not entering a trial (e.g., in screening logs) will also lend some transparency to trial conduct. These reported details will need to go beyond ambiguous statements such as “clinician discretion”. Another alternative is for nurses or other professionals to undertake recruitment. While nurses do increasingly take on this responsibility, clinicians need to be accustomed to presenting equipoise and uncertainty comfortably: patients will still look to clinicians for advice, irrespective of whether clinicians are actively recruiting into an RCT. Furthermore, there is encouraging emerging evidence that it is possible for clinicians to learn recruitment skills [22,44,45].

This study found that clinicians often shared their opinions and predictions about RCT outcomes with patients. This raises questions around what should be expected of clinicians who hold the dual roles of physician and RCT recruiter. Avoiding personal views may be difficult in situations where patients request an opinion or recommendation. Trials that involve treatments that differ to usual care pose particular challenges, as clinicians need to have a degree of faith in “the new” to justify disrupting usual practice. Conversely, clinicians may also need to justify use of older, less innovative treatments in trials that evaluate novel interventions that have already diffused into routine practice without a supporting evidence base. Consistently communicating the concept of community equipoise throughout the appointment is one potential solution to responding to recommendation requests. The principles underpinning Chard and Lilford’s distinction between absolute equipoise and effective equipoise may also be useful here, in which equipoise around a single outcome (absolute equipoise) is distinguished from equipoise created by balancing multiple considerations (e.g., financial costs, side effects, effectiveness, quality of life) (effective equipoise) [5]. This alternative approach to viewing equipoise may provide a helpful framework in practice for balancing the advantages and disadvantages of trial treatments in efforts to sustain equipoise.

The common occurrence of particular equipoise-related difficulties across trials suggests that these challenges are amenable to generic training that could be incorporated into undergraduate and postgraduate medical curricula—particularly given that clinicians are increasingly expected to discuss issues of uncertainty and evidence. Recommendations for improving communication may also be adopted if clinicians are able to consider and apply these in the context of an RCT they are working within. A current programme of applied research has been developed to address recruitment difficulties by delivering tailored interventions as RCTs proceed [22]. Interventions applied within RCTs to date have included training around some of the issues reported in the present study [45]. Our findings also raise the possibility of targeting training to recruiters based on their individual perceptions of equipoise expressed at the trial’s outset (e.g., through interviews). Further research will need to investigate whether training can lead to sustained change in the practices of less balanced clinicians.

This study did not intend to make causal inferences between recruiters’ practices and trial participation outcomes, though there is potential for future research to examine possible associations. There is also potential to consider how categorisation of recruiters as more or less balanced is associated with recruitment outcomes, although this would run counter to our interpretation of individual equipoise falling on a continuum. Such efforts will need to consider the complex nature of patient decision-making, which can be influenced by an array of factors within and outside the recruitment appointment. Future research may also consider patients’ interpretations of appointment events—particularly their perspectives on equipoise and trial participation immediately following appointments, and levels of informed consent.

### Conclusion

Communicating equipoise in practice is a delicate process that can be challenging, especially when clinicians are negotiating their own struggles with equipoise. Despite clinicians’ assumptions that personal biases and preferences can be set aside, these can unwittingly materialise in encounters with patients. This phenomenon calls for careful reflection and training to enable clinicians to communicate equipoise as they intend. Guidelines may need to consider the possibility of offering training based on clinicians’ conveyed (rather than intended) perceptions of equipoise.

## Supporting Information

S1 Data Interview extracts illustrating evidence of 13 clinicians’ less balanced views. (DOCX)

S1 Table Consolidated Criteria for Reporting Qualitative Research (COREQ) completed checklist. (DOCX)

S1 Text Details of ethical approval. (DOCX)

S2 Text COREQ checklist additional information. (DOCX)

## Abbreviations

QRI: Qualitative Research Integrated within Trials Recruitment Intervention
QuinteT: Qualitative Research Integrated within Trials
RCT: randomised controlled trial
