# Supplementary material for: Conveying Equipoise during Recruitment for Clinical Trials: Qualitative Synthesis of Clinicians’ Practices across Six Randomised Controlled Trials
Source: PLoS Med. 2016 Oct 18;13(10):e1002147. doi: 10.1371/journal.pmed.1002147 (PMC5068710; doi:10.1371/journal.pmed.1002147)
Supplement: S1 Data — (DOCX) [file pmed.1002147.s001.docx]

**S1 Data**

**Interview extracts illustrating evidence of 13 clinicians’ ‘less balanced’ views.**

**RCT1, R2**

R2: So I’m struggling personally with what we’re going to do in the trial with the patients who for whatever reason don’t get on with [treatment y].

R2: I hope that I can do it with sufficient equipoise. The truth is when someone says “I want to have [treatment y],” I do sometimes… my heart sinks, because I think this patient is not going to do well with [treatment y] and I sometimes feel for them having [treatment y] on the NHS, well I know the NHS will fall down, in so many ways for reasons we’ve just described.

Interviewer: You’ve said quite a few times that you think you are in equipoise as such.

R2: I’m hoping I’m in equipoise […]. Well I’m probably I’m doing my best to be in equipoise]

R2: I think that the quality of life with [treatment x] would be better than [treatment y] and I think I can accept that the average weight loss with [treatment y] can be the same as [treatment x], my worry is the patient who fails with [treatment y]. And all the evidence suggest that the proportion of those patients will be much higher for [treatment y] than [treatment x], so my worry will be what to do with those patients. So the, the primary end point (will be) the same, but the quality of life will be poorer for [treatment y].

**RCT1, R4**

R4: Hmm I have to be say, I mean, being honest, with the more elderly patients with more co-morbidities hmm with the diabetes, less mobile, I tend to go straight for [treatment x] on those patients. Just because they’ve got a limited life expectancy, to get their co-morbidities under control a bit better.

R4: In the younger patients I get with the less co-morbidities, I certainly have a preference for [treatment y] rather than [treatment x].

R4: I think we’ll see what other trials have demonstrated. I think we’ll see that the weight loss in hmm [treatment x] is quicker initially but then there may be weight regain long term, you know, if the intensity of the follow-up in [treatment y] patients is high, then their weight loss would be good, hmm and they may start to hmm merge into one group of patients at about 5 years. If [treatment y] follow-up wasn’t good, then [treatment y] group would tend to fail and then it would separate the 2 groups of patients, with [treatment x] doing better long term.

Interviewer: Ok, right ok. Do you see yourself if you were a patient, would you agree to be randomised into a trial like this?

R4: With my current knowledge, no (laughs).

**RCT2, R7**

R7: My gut reaction is that probably [treatment x], there is no reason to think that [treatment x] won’t do something because it works in metastatic disease. We don’t have the data for [treatment x] in bladder cancer you know it’s been neo adjuvant treatment but your gut reaction is probably that it does something.

**RCT3, R8**

Interviewer: So yeah, I was going to ask whether you had a hunch about the outcome of the trial?

R8: I think there will be no difference between [technology] driven treatment [treatment group x] and treatment for everyone [treatment group y]

Interviewer: Okay so there’s going to be no difference between the groups. So what would be the implications of that?

R8: So if the [technology] works, the outcome between the two groups should be exactly the same.

**RCT3, R10**

R10: What do I think? I… I think it would be hard pushed to show a difference. Because I don’t think the [technology] will be discriminating enough to… really… really show the difference between the groups.

Interviewer: OK. In terms of survival rates?

R10: Yeah.

Interviewer: Which would be a good thing?

R10: Yeah. It would. Yeah. Yeah.

R10: I mean I think it’s quite generous the… the high end (of the eligibility criteria). So some of the patients with the higher end of the lymph node involvement…I’d be nervous about not giving them [treatment y]. But I have to say I’m fairly confident if they had the [technology] [treatment group x] they’d probably end up having [treatment y] anyway because they’d probably come out at high risk.

Interviewer: That leads quite nicely to my next question; if you were kind of… it’s difficult, but do you feel you would have a treatment preference if you were a patient?

R10: Yeah I mean I think in fairness if… if there’s lots of lymph node involvement I would go for [treatment y]. And I don’t know… what my limit would be. Certainly more than four. And if there was three, two… I don’t know. One… one lymph node…I’d do the [technology]- I’d do the trial.

Interviewer: OK. Yeah. That’s interesting. But four or anything over four you’d feel that…

R10: I think above four I’d have [treatment y]

Interviewer: Any reasons? What do you feel you would…?

R10: I guess because it seems to be reflective - the lymph node involvement - reflective of the risk of distant recurrence. And… you know, four is a substantial amount. It’s an arbitrary figure, but we use four for radiotherapy (inaudible), if it’s four or more you might give… maybe refer for radiotherapy (inaudible).

**RCT3, R11**

R11: As I say, I think to recruit patients and to get people through the trial whoever’s doing that needs to generally believe in the existing evidence and believe that the [technology] is likely to be a useful, valid tool in the future and that there may be some people who don’t believe that or who need more persuading if that’s the case, so I think really from the potential recruiter’s point of view, I think being very clear about the evidence that already exists to support the [technology] is important in trying to get recruiters on board for the wider study.

**RCT4- R12**

R12: Personally, I think that [treatment x] will be the way forward for asymptomatic disease in the future, so I think that they may well be getting the treatment of the future in advance.

**RCT5- R16**

R16: So, I think as I said at the beginning, I believe that [treatment x] or aspects of [treatment x] probably do work, but it’s like all operations. The key thing is if the right operation is done in the right patient for the right problem then I think you’ll get a good outcome. So I think if this study – I think this study may identify the people who have fulfilled certain inclusion criteria, I think probably the outcome will be that [treatment x] does work. So there you go, I’ve said it!

**RCT5, R17**

R17: Patients who are potentially eligible for the trial, I would tell them that we are undertaking the study, because we are uncertain as to exactly why and how [treatment x] produces its effect. […] The intervention seems to work for the majority. Because of that, we are looking to study to see whether it is the mechanical part of [treatment x] that is producing improvement or not. One way of doing that is this study. I am very comfortable with having my patients in that study, because we do not understand why [treatment x] works.

Interviewer: Yes. Have you got a sense as to what the outcome of the trial will be? Do you think it will say that [treatment x] does make the difference, or?

R17: I suspect it will do. I suspect it will. Because just intuitively, if people have a pure diagnostic undertaking, they are likely to be through any post-surgical discomfort rather more rapidly, and are more likely not to take as seriously any rehabilitation requirements, for want of a better phrase. Whereas people that have had [treatment x], will have that post-surgical discomfort for a longer period, to remind them to stick with the programme. But that is purely intuitive.

**RCT6, R18**

R18: I have to trust myself not let my prejudices get in the way, and you have to… as a clinician that is so, so devoted to the patient (unclear) I, I’m motivated by outcome, I really am devoted, and so I don’t allow myself to do that.

**RCT6 R19**

R19: Hmm I, I think that the (pause)… yeah so we all sit there in MDT saying you know as far as we know the outcomes of [treatment y] for this patient is likely to be the same as for [treatment x] and we all say that, and I think we all believe that probably… hmm I think we all believe that. It is the case though that historically because we’ve had such a strong surgical lead to the MDT we’ve got a long history of [treatment x] for that group of patients. That, for a long time, has been our default setting, and that standard of care has been [treatment x] for these patients […]. The gold standard has always been [treatment x] for that group of patients because we don’t believe it’s any worse, hmm and we’ve got, we’ve got (name of consultant) has got very good data for the outcomes for that group of patients. I think if anything we’ve got less data for [treatment y] than we have for [treatment x]. […] Unless we’ve got good reason to change to [treatment y], I think it will remain our standard,

R19: I think (we) probably believe and hope that [treatment y] is the equivalent to [treatment x] and that’s what the data that we’ve got would suggest, but of course we haven’t got a big randomised trial to prove that. Until we have, we can’t be sure that one’s equivalent to the other, but at least we know pretty well how good [treatment x] is.

**RCT6, R20**

R20: I’m a non-surgeon. I personally would lean to [treatment y], but I would love to have the evidence. So yeah I would probably lean to it, but I would try and keep my conversation just straightforward.

**RCT6, R22**

Interviewer: And over the years has your opinion changed about which is a preferential treatment?

R22: Um… has it changed? Well I don’t really know. I probably would have been biased towards a [treatment x] a few years ago. I’m probably biased towards a [treatment x] slightly, but I try not to express that. Whether I succeed or not I don’t know.

R22: I would like to know what the local recurrence rates are so I would possibly slightly worry about having [treatment y]. I’ve seen- I’ve seen lots of patients who’ve had [treatment y] who it looks quite good and then- then they start to get a stricture…
