# Supplementary material for: Conveying Equipoise during Recruitment for Clinical Trials: Qualitative Synthesis of Clinicians’ Practices across Six Randomised Controlled Trials
Source: PLoS Med. 2016 Oct 18;13(10):e1002147. doi: 10.1371/journal.pmed.1002147 (PMC5068710; doi:10.1371/journal.pmed.1002147)
Supplement: S1 Text — (DOCX) [file pmed.1002147.s003.docx]

**S1 Text**

**Details of ethical approval**

Ethical approval to conduct this research was secured through each individual RCT. The research ethics committee names, reference numbers, and dates of approval for the recruitment sub-study in each RCT are as follows (in alphabetical order):

*Acst-2: Yorkshire & The Humber - South Yorkshire Research Ethics Committee (13/YH/0409), November 2013.*

*By-Band-Sleeve: South West - Frenchay Research Ethics Committee (11/SW/0248), December 2011.*

*CSAW: South Central–Oxford B Research Ethics Committee (12/SC/0028), February 2012.*

## *Feasibility RCT of definitive chemoradiotherapy or chemotherapy and surgery for oesophageal squamous cell cancer: North Somerset and South Bristol Research Ethics Committee (09/H0106/69), October 2009.*

*POUT: North West - Greater Manchester South Research Ethics Committee (11/NW/0782),*

*December 2011*

*Optima prelim: South East Coast Surrey Research Ethics Committee (12/LO/0515), June 2012*
