# Supplementary material for: Conveying Equipoise during Recruitment for Clinical Trials: Qualitative Synthesis of Clinicians’ Practices across Six Randomised Controlled Trials
Source: PLoS Med. 2016 Oct 18;13(10):e1002147. doi: 10.1371/journal.pmed.1002147 (PMC5068710; doi:10.1371/journal.pmed.1002147)
Supplement: S2 Text — (DOCX) [file pmed.1002147.s004.docx]

**S2 Text**

**COREQ checklist additional information**

**Biographies of researchers conducting qualitative data collection and analyses**

Leila Rooshenas (LR), Daisy Elliott (DE), Marcus Jepson (MJ), Sangeetha Paramasivan (SP), Caroline Wilson (CW), and Jenny Donovan (JLD) are all health services researchers, hold PhDs in health services research, and had at least 3 (WTE) years of post-doctoral qualitative research experience at the time of interviews. All bar MJ are female. All bar JLD were post-doctoral research associates, senior research associates, or fellows at the time of study conduct. Sean Strong (SS) was a male surgical trainee and doctoral student at the time of data collection, and was conducting his first qualitative research study on the impact of team work on RCT recruitment (under the supervision of SP and JLD).

JLD was a professor of social medicine at the time of data collection. JD conducted one interview with a clinical recruiter, and shared a similar role to the individual she was interviewing; both JD and the interviewee were co-applicants and TMG members for the RCT in question. JLD was also a chief investigator of an ongoing multi-centre pragmatic RCT (the ProtecT study) at the time of this study. This may have influenced the dynamics of the interview.

With exception to JLD, all the named researchers involved in qualitative data collection/analysis approached this project as naive researchers, with little knowledge of the field or literature.

We have no reason to believe that any of the researchers’ personal or research experiences would have influenced their approaches to data collection and analysis in a noteworthy way.

**Contact with study participants**

None of the researchers conducting data collection or analysis had prior contact or relationships with the research participants prior to the interviews. The only exception to this was JLD, who was part of the same TMG as the individual she was interviewing (although the RCT has not yet begun). Some of the researchers had developed professional working relationships with some of the clinicians at the time of audio-recording appointments, though any contact was in relation to trial conduct or the audio-recording process itself.

Research participants all received study information sheets in advance of data collection processes, which explained why the research was being undertaken. Information sheets included no personal details about the researchers or their personal aspirations. As such, interview participants will not have been informed of any personal information about the researchers prior to being interviewed, although they will have known the identity of the researchers (through email correspondence), and thus had opportunity to read about researchers’ research interests in advance (e.g. via the internet). Although this was not explicit in interviews, there is a possibility that participants’ prior knowledge of researchers’ interests influenced their accounts and responses.
